# Prevalence of Physical Activity in the United States: Behavioral Risk Factor Surveillance System, 2001

**Published:** 2005-03-15

**Authors:** Caroline A Macera, Sandra A Ham, Michelle M Yore, Deborah A Jones, C Dexter Kimsey, Harold W Kohl, Barbara E Ainsworth

**Affiliations:** Graduate School of Public Health, San Diego State University; Centers for Disease Control and Prevention, Division of Nutrition and Physical Activity, Atlanta, Ga; Centers for Disease Control and Prevention, Division of Nutrition and Physical Activity, Atlanta, Ga; Centers for Disease Control and Prevention, Division of Nutrition and Physical Activity, Atlanta, Ga; Centers for Disease Control and Prevention, Division of Nutrition and Physical Activity, Atlanta, Ga; Centers for Disease Control and Prevention, Division of Nutrition and Physical Activity, Atlanta, Ga; San Diego State University, Department of Exercise and Nutritional Sciences, San Diego, Calif

## Abstract

**Introduction:**

The health benefits of regular cardiovascular exercise are well-known. Such exercise, however, has traditionally been defined as vigorous physical activity, such as jogging, swimming, or aerobic dance. Exercise of moderate intensity also promotes health, and many U.S. adults may be experiencing the health benefits of exercise through lifestyle activities of moderate intensity, such as yard work, housework, or walking for transportation. Until recently, public health surveillance systems have not included assessments of this type of physical activity, focusing on exercise of vigorous intensity. We used an enhanced surveillance tool to describe the prevalence and amount of both moderate-intensity and vigorous-intensity physical activity among U.S. adults.

**Methods:**

We analyzed data from the 2001 Behavioral Risk Factor Surveillance System, a state-based, random-digit–dialed telephone survey administered to U.S. adults aged 18 years and older (n = 82,834 men and 120,286 women). Physical activity behavior was assessed using questions designed to quantify the frequency of participation in moderate- or vigorous-intensity physical activities performed during leisure time or for household chores and transportation.

**Results:**

Overall, 45% of adults (48% of men and 43% of women) were active at recommended levels during nonworking hours (at least 30 minutes five or more days per week in moderate-intensity activities, equivalent to brisk walking, or at least 20 minutes three or more days per week in vigorous activities, equivalent to running, heavy yard work, or aerobic dance). Less than 16% of adults (15% of men and 17% of women) reported no moderate or vigorous activity in a usual week.

**Conclusion:**

Integrating surveillance of lifestyle activities into national systems is possible, and doing so may provide a more accurate representation of the prevalence of recommended levels of physical activity. These results, however, suggest that the majority of U.S. adults are not active at levels associated with the promotion and maintenance of health.

## Introduction

The 1996 Surgeon General's report on physical activity and health (1) emphasized the health benefits of moderate-intensity physical activities, especially everyday activities. These activities include heavy yard work, brisk walking, and housework in addition to purposeful leisure-time exercise. Participation in activities of at least moderate intensity is associated with numerous health benefits, including lower all-cause mortality, lower cardiovascular mortality, improved function, and enhanced quality of life. Although vigorous-intensity activities (such as running and other aerobic sports) that challenge the cardiovascular system are strongly related to many positive health outcomes, less than 15% of the U.S. population is active at that level, and this prevalence did not change from 1990 to 1998 (2). Several organizations and agencies have supported health-related recommendations of 30 minutes per day of moderate-intensity physical activities on most days of the week (3,4), but this level of physical activity has been difficult to track in the U.S. population.

Historically, surveillance systems for physical activity were designed to measure leisure-time activities with an emphasis on participation in vigorous-intensity sports. They did not assess participation in lifestyle physical activities of moderate intensity that might be related to household, transportation, or occupational activities. Therefore, it is not possible with historical surveillance systems to know how many Americans have been achieving a level of physical activity to ensure health benefits through a broader range of physical activities that occur during nonworking hours. To address this question, we recently documented the prevalence of physical activity during nonworking hours for each state in the United States (5). The purpose of this paper is to extend these findings by describing the epidemiology of physical activity recommendations during nonworking hours for U.S. adults.

## Methods

The Behavioral Risk Factor Surveillance System (BRFSS) is a population-based, random-digit–dialed telephone survey administered to U.S. civilian, noninstitutionalized adults aged 18 years and older in the 50 states and the District of Columbia. Questions on physical activity have been included in most years since the survey began in 1984. Between 1997 and 2000, the Physical Activity and Health Branch at the Centers for Disease Control and Prevention developed a new set of questions designed to measure occupational, household, and leisure-time physical activity with a special emphasis on moderate-intensity activities. Questions were validated using activity logs and accelerometers and subsequently modified (6). Additional testing included cognitive testing in 1998 and 1999 and a pilot test in four states (Nebraska, Georgia, Hawaii, and Michigan) in 1999. Questions were modified to reflect changes suggested by the various tests, and because of space constraints, a subset of the questions was implemented in the 2001 BRFSS.

The final questionnaire included items about moderate and vigorous activities that are performed during nonworking hours in a usual week (5). The questions included the number of days per week and number of minutes per day. These questions required the respondent to self-select the intensity of an activity, whereas in previous BRFSS surveys the participant specified an activity and standard intensity values were applied according to the respondent's age and sex. Both approaches generate useful measurements, but the self-assessed intensity method was selected because of the wide individual variation in fitness and energy expenditure required to perform a particular activity. A table comparing the questions used in the BRFSS for 2000 and 2001 has been previously published (5).

In addition to questions on moderate and vigorous activities, a single item was asked of all employed persons. This item classified occupational activity as "mostly sitting or standing," "mostly walking," or "mostly heavy labor."

The criteria for determining compliance with health-related physical activity guidelines were adapted from the Surgeon General's report on physical activity and health (1) and other consensus statements (3,4). Respondents were classified as meeting recommendations if they reported participation in moderate-intensity activities on five or more days per week for 30 or more minutes per day and/or vigorous activity for three or more days per week for 20 minutes or more per day. Respondents were classified as inactive if they reported no moderate or vigorous physical activity on any day during a usual week.

In addition to employment activity status, demographic variables included were age, educational level (less than high school, high school graduate, some college, and college graduate), race/ethnicity (non-Hispanic white, non-Hispanic black, Hispanic, other), body mass index (BMI calculated as weight [kg]/height [m]^2^), and region of the country. BMI was categorized into underweight (<18.5), healthy weight (18.5–24.9), overweight (25.0–29.9), and obese (⩾30.0). Region of the country was defined as follows: Midwest (Illinois, Indiana, Iowa, Kansas, Michigan, Minnesota, Missouri, Nebraska, North Dakota, Ohio, Oklahoma, South Dakota, Wisconsin); Northeast (Connecticut, Maine, Massachusetts, New Hampshire, New Jersey, New York, Pennsylvania, Rhode Island, Vermont); South (Alabama, Arkansas, Delaware, District of Columbia, Florida, Georgia, Kentucky, Louisiana, Maryland, Mississippi, North Carolina, South Carolina, Tennessee, Texas, Virginia, West Virginia); and West (Alaska, Arizona, California, Colorado, Hawaii, Idaho, Montana, Nevada, New Mexico, Oregon, Utah, Washington, Wyoming).

All data were stratified by sex, and all prevalence estimates were age-adjusted to the year 2000 standard population. SUDAAN statistical software (Research Triangle Institute, Research Triangle Park, NC) was used to adjust for the complex sample survey design. Logistic regression models were calculated using "meeting physical activity recommendations" as the outcome.

## Results

After exclusion of 7370 observations from Guam, Puerto Rico, and the Virgin Islands, the analysis sample included 203,120 respondents (82,834 men and 120,286 women). The median response rate for all of the states included in the 2001 BRFSS was 51.1% (7). The method used to calculate the response rate was based on a formula developed by the Council of American Survey Research Organizations (CASRO) and reflects the efficiency of telephone sampling as well as the degree of cooperation among the eligible respondents contacted (8). Data were weighted by age and sex to reflect each state's most recent estimate of the adult population. 

The distributions of age, educational level, occupational status, and other variables for men and women in the sample are shown in Table 1. Sixty-four percent of the sample was aged 30 to 64 years. Eleven percent had less than a high school education, while 30% had graduated from college. The distributions of age and education were similar for men and women. Overall, 38% of the respondents were not currently employed (29% of men and 43% of women), and 41% of men and women had jobs that required mostly sitting or standing. 

Overall, 45% of the respondents were active at the recommended levels in their nonworking hours (48% of men and 43% of women) (Table 2). The prevalence of meeting the criteria for moderate activity was similar for both men and women (32%), but men surpassed women in meeting the criteria for vigorous activity (29% for men vs 20% for women). The data indicate that 13% of men and 8% of women met the guidelines for both moderate and vigorous activity, while only 16% of the respondents (15% of men and 17% of women) were inactive (no moderate or vigorous activity at any time during a usual week).

As expected, the prevalence of meeting recommended levels of physical activity was generally lower at older ages. The difference between the youngest (18 to 29 years) group and oldest (⩾75 years) group in meeting recommendations was slightly greater among women than men: 50% of women aged 18 to 29 vs 27% of women aged 75 or older, and 58% of men aged 18 to 29 vs 38% for men aged 75 or older. Also as expected, for both men and women, the prevalence of recommended activity was higher among non-Hispanic whites than non-Hispanic blacks, Hispanics, or "other" racial/ethnic groups. Meeting recommended levels of physical activity was successively higher with greater educational attainment for both men and women. 

The prevalence of recommended physical activity varied by BMI, with about half the men classified as healthy weight or overweight meeting recommended levels, while fewer obese or underweight men did so. For women, 50% of those in the healthy weight group met recommended levels vs only 33% of obese women. Regional differences were noted; the West had the highest prevalence of recommended physical activity for both men and women. As for employment status, women who were active on the job (mostly walking or heavy labor) were more active during nonworking hours than those who were less active on the job or the unemployed. For men, those doing mostly heavy labor were more active during nonworking hours than other groups.

Odds ratios for meeting recommendations for moderate or vigorous activity are shown in Table 3 by age, race/ethnicity, education, BMI, region, and occupational activity. In both sexes, younger adults (aged 18 to 29) were more active than older adults, and non-Hispanic whites were more active than the other racial/ethnic groups. Also, for both men and women, activity was higher among those with at least a high school education than among those who did not finish high school. Both the obese and the underweight groups were less active than the healthy weight group. In both sexes, those who were active at work (walking or heavy labor) were more active during nonworking hours than those who mostly sat or stood at work.

## Discussion

Because emerging research in the past 15 years has indicated a dose–response relationship between physical activity and health as well as the specific health benefits of moderate-intensity physical activity, surveillance systems must be able to document prevalence and trends for moderate-intensity lifestyle activity. The surveillance system for physical activity used in the 2001 BRFSS broadens the concept of physical activity beyond traditional sports-related vigorous exercise by including examples of housework and yard work. Although these questions provide a more complete picture of the prevalence of health-related physical activity than those previously used, other domains, such as transportation and childcare activities, which are not mentioned in examples, may also account for activity that is not easily remembered or reported. Future work in this area should attempt to quantify all domains so that surveillance systems can monitor and track patterns of lifestyle physical activity.

The National Health Interview Survey (NHIS) measures moderate- and vigorous-intensity leisure-time physical activity for national *Healthy People 2010* objectives. However, because of the sampling frame, it is not feasible to generate state-specific estimates of physical activity prevalence using NHIS data. Previous work has shown that state-specific BRFSS data can be weighted and combined to produce prevalence estimates of smoking and alcohol use comparable to national surveys (9). However, the prevalence estimates of physical activity generated by NHIS and combined BRFSS data will be different because of slight changes in question wording that have been shown to affect prevalence (10). In addition, BRFSS can be used by states, some metropolitan areas, and some counties to monitor progress toward the *Healthy People 2010* objectives for reducing the proportion of adults who engage in no leisure-time physical activity as well as increasing the proportion of adults who engage in regular physical activity of moderate and/or vigorous intensity.  

Although the prevalence of U.S. adults achieving recommended levels of physical activity was higher in 2001 (45.4%) than in 2000 (26.2%) (5), this finding was expected because of the addition of nonsports-related examples (such as heavy yard work and housework). Changes in surveillance systems are often difficult to make and can result in losing the ability to track temporal trends. The 2001 survey, however, also included a tracking question that had been used before 2001: "During the past 30 days, other than your regular job, did you participate in any physical activity or exercise such as running, calisthenics, golf, gardening, or walking for exercise?" The prevalence of inactivity as measured by this question did not change much from 2000 to 2001 (27.4% to 26.0%), suggesting that the increases seen in recommended activity (from 26% in 2000 to 45% in 2001) may be primarily because of the expanded definition of physical activity and the inclusion of the additional examples of yard work and housework (5). Recent data based on 35 states with physical activity data from 1988 to 2002 indicate that the prevalence of physical inactivity continues to slowly decrease (25.1% in 2002), which may suggest that recommended physical activity may increase over time (11).

BRFSS has some limitations. First, it is a telephone-based system that surveys noninstitutionalized adults residing in the United States and is thus limited in its ability to capture people without telephones or those who do not reside at home. Second, all information is self-reported and subject to potential misclassification bias. Respondents may be prone to providing socially desirable answers. Third, the statistical issues involved in combining data from state-specific surveys may have influenced estimates of the prevalence of physical activity.

It is notable that even with expanded definitions of physical activity, less than half of the U.S. adult population is achieving sufficient activity to obtain health benefits. Although the recommended levels of physical activity as defined here are associated with health benefits, these are minimal amounts recommended for adults of all ages; a fully active lifestyle would include aerobic activities as well as those that increase strength and flexibility, which were not measured in this study. Members of the U.S. Preventive Services Task Force have recently reviewed the literature and identified several effective interventions that were shown to increase physical activity among U.S. adults and adolescents (12). Among the recommended interventions are point-of-decision prompts to encourage stair use, social support for physical activity in community settings, individually adapted health behavior change, and creation of places for physical activity combined with informational outreach activities. To more fully understand the nature of physical activity in the population and to assess changes at the population level that may result from suggested interventions, future surveillance systems will need to capture purposeful physical activity (such as stair climbing) that is not usually of a duration to warrant reporting (at least 10 minutes).

In summary, less than half of U.S. adults meet minimal physical activity recommendations, even with more inclusive methods of surveillance that include some lifestyle activities. Even so, this study identified predictable population differences that help point the way for population-based promotion efforts.

## Figures and Tables

**Table 1:** Percent Distribution of Age, Education, and Other Variables for Men and Women, Behavioral Risk Factor Surveillance System, United States, 2001

	**Men, % (n=82,834)**	**Women, % (n=120,286)**	**Total, % (N=203,120)**

**Age, years**			

18-29	17.9	16.3	17.0
30-44	31.8	30.6	31.1
45-64	33.7	32.4	32.9
65-74	10.1	11.0	10.6
⩾75	6.5	9.8	8.4

**Education**			

Less than high school	11.1	11.5	11.3
High school graduate	30.8	32.0	31.5
Some college or technical school	25.3	28.6	27.3
College graduate	32.8	28.0	29.9

**Race/ethnicity**			

Non-Hispanic white	80.1	79.0	79.5
Non-Hispanic black	6.7	8.7	7.9
Hispanic	6.3	6.4	6.4
Other	6.9	5.9	6.3

**Body mass index (BMI)**			

Underweight (<18.5)	0.9	3.0	2.1
Healthy weight (18.5-24.9)	31.9	46.6	40.4
Overweight (25.0-29.9)	45.6	29.5	36.3
Obese (⩾30.0)	21.7	21.0	21.3

**Region**			

South	30.8	33.1	32.1
Midwest	23.3	23.3	23.3
Northeast	22.4	22.2	22.3
West	23.5	21.3	22.2

**Employment status/occupational activity**			

Employed, mostly sitting or standing	40.7	40.7	40.7
Employed, mostly walking	14.5	12.2	13.1
Employed, mostly heavy labor	15.7	3.9	8.7
Not currently employed	29.2	43.3	37.5

**Table 2:** Age-Adjusted Prevalence of Physical Activity Status by Sex, Behavioral Risk Factor Surveillance System, United States, 2001 (N=203,120)

**Demographic Group**	**Inactive^a^% (SE)**	**Moderate^b^% (SE)**	**Vigorous^c^% (SE)**	**Recommended^d^% (SE)**
**Overall**	15.9 (0.15)	31.6 (0.18)	24.3 (0.17)	45.4 (0.2)

**Men**				

**Age, years**				

All ages	15.0 (0.22)	31.5 (0.28)	29.2 (0.28)	47.9 (0.31)
18-29	10.3 (0.48)	35.7 (0.68)	43.3 (0.73)	57.8 (0.74)
30-44	11.8 (0.37)	30.3 (0.48)	31.9 (0.50)	48.3 (0.55)
45-64	16.3 (0.40)	29.5 (0.48)	23.7 (0.45)	43.4 (0.54)
65-74	21.4 (0.80)	34.1 (0.87)	18.4 (0.90)	45.7 (1.01)
⩾75	29.7 (1.05)	29.2 (1.00)	11.5 (0.74)	38.4 (1.13)

**Race/ethnicity**				

Non-Hispanic white	13.0 (0.22)	34.3 (0.31)	30.6 (0.30)	50.6 (0.33)
Non-Hispanic black	20.8 (0.90)	22.5 (0.87)	26.2 (0.92)	40.3 (1.05)
Hispanic	21.8 (1.19)	24.6 (1.13)	26.5 (1.51)	41.6 (1.52)
Other	17.0 (1.04)	27.8 (1.19)	25.8 (1.12)	43.1 (1.38)

**Education**				

Less than high school	29.3 (0.94)	23.0 (0.82)	19.6 (0.90)	35.6 (1.04)
High school	18.1 (0.44)	31.6 (0.50)	25.1 (0.47)	46.0 (0.57)
Some college or technical school	12.6 (0.40)	34.4 (0.55)	30.0 (0.53)	50.3 (0.59)
College graduate	8.6 (0.32)	33.1 (0.53)	35.6 (0.54)	52.7 (0.56)

**Body mass index (BMI)**				

Underweight (<18.5)	19.0 (1.41)	29.7 (1.61)	27.7 (1.61)	45.6 (1.82)
Healthy weight (18.5-24.9)	13.7 (0.39)	33.2 (0.49)	31.7 (0.50)	50.4 (0.55)
Overweight (25.0-29.9)	13.1 (0.33)	33.4 (0.44)	31.2 (0.47)	50.8 (0.51)
Obese (⩾30.0)	18.2 (0.51)	26.8 (0.60)	22.7 (0.58)	40.2 (0.67)

**Region**				

South	17.8 (0.36)	32.4 (0.61)	26.9 (0.40)	45.4 (0.46)
Northeast	15.1 (0.52)	32.1 (0.53)	29.6 (0.59)	48.9 (0.68)
Midwest	15.0 (0.43)	29.0 (0.41)	28.9 (0.52)	47.9 (0.58)
West	10.7 (0.52)	34.2 (0.74)	32.6 (0.80)	51.1 (0.85)

**Employment status/occupational activity**				

Employed, mostly sitting or standing	12.1 (0.46)	28.8 (0.51)	30.6 (0.50)	46.9 (0.58)
Employed, mostly walking	14.4 (0.73)	33.1 (0.93)	26.8 (0.77)	47.0 (1.03)
Employed, mostly heavy labor	16.4 (1.07)	37.0 (1.03)	33.0 (0.97)	53.0 (1.17)
Not currently employed	22.8 (0.68)	31.4 (0.67)	24.6 (0.68)	45.3 (0.77)

**Women**				

**Age, years**				

All ages	16.7 (0.20)	31.8 (0.23)	19.6 (0.20)	43.0 (0.26)
18-29	11.8 (0.41)	34.7 (0.59)	28.9 (0.56)	49.8 (0.63)
30-44	11.9 (0.31)	34.5 (0.42)	22.7 (0.37)	46.5 (0.46)
45-64	17.0 (0.39)	31.0 (0.40)	16.4 (0.33)	40.7 (0.45)
65-74	24.5 (0.73)	28.0 (0.65)	9.5 (0.42)	36.1 (0.75)
⩾75	39.6 (0.83)	20.5 (0.63)	5.7 (0.39)	26.9 (0.75)

**Race/ethnicity**				
Non-Hispanic white	13.2 (0.18)	34.5 (0.26)	21.5 (0.23)	46.0 (0.28)
Non-Hispanic black	28.4 (0.73)	21.2 (0.64)	14.3 (0.55)	31.4 (0.74)
Hispanic	27.1 (1.03)	26.2 (0.88)	14.2 (0.67)	35.6 (1.01)
Other	19.1 (1.14)	29.4 (1.16)	18.7 (0.92)	41.2 (1.32)

**Education**				

Less than high school	32.3 (0.83)	26.5 (0.77)	10.5 (0.56)	34.0 (0.87)
High school	18.8 (0.36)	30.3 (0.40)	15.8 (0.33)	40.3 (0.45)
Some college or technical school	14.0 (0.34)	32.8 (0.43)	20.6 (0.38)	44.3 (0.47)
College graduate	9.9 (0.35)	35.7 (0.47)	26.4 (0.43)	49.2 (0.52)

**Body mass index (BMI)**				

Underweight (<18.5)	19.8 (0.98)	32.9 (1.10)	21.2 (0.99)	43.6 (1.20)
Healthy weight (18.5-24.9)	12.8 (0.26)	36.5 (0.35)	25.2 (0.32)	49.9 (0.38)
Overweight (25.0-29.9)	15.7 (0.38)	31.2 (0.46)	17.6 (0.39)	41.8 (0.51)
Obese (⩾30.0)	22.2 (0.51)	25.6 (0.54)	11.4 (0.39)	32.8 (0.59)

**Region**				

South	19.8 (0.29)	32.5 (0.52)	17.8 (0.29)	39.7 (0.37)
Northeast	16.8 (0.45)	32.0 (0.44)	20.5 (0.45)	44.6 (0.58)
Midwest	15.5 (0.36)	28.7 (0.33)	19.5 (0.40)	42.8 (0.49)
West	13.0 (0.54)	36.0 (0.63)	22.1 (0.52)	47.2 (0.69)

**Employment status/occupational activity**				

Employed, mostly sitting or standing	12.8 (0.43)	30.7 (0.46)	20.3 (0.35)	42.3 (0.51)
Employed, mostly walking	15.7 (1.06)	33.1 (0.85)	21.0 (0.62)	44.2 (0.98)
Employed, mostly heavy labor	12.9 (1.34)	40.4 (1.76)	28.2 (1.69)	55.6 (1.80)
Not currently employed	20.5 (0.35)	33.1 (0.42)	17.6 (0.34)	43.1 (0.46)

a Inactive = no moderate or vigorous activity.

b Moderate = participated in 30 minutes per day of moderate-intensity activity on five or more days per usual week.

c Vigorous = participated in 20 minutes per day of vigorous-intensity activity on three or more days per usual week.

d Recommended = met moderate or vigorous recommendations or both. Note that these two categories are not mutually exclusive.

**Table 3:** Adjusted Odds Ratios for Meeting Recommended Levels of Physical Activity Among Men and Women, Behavioral Risk Factor Surveillance System, United States, 2001^a^

	**Men (n=82,834)**	**Women (n=120,286)**

	**OR (95% CI)**	**OR (95% CI)**

**Age, years**		

18-29	2.33 (2.06-2.64)	2.77 (2.51-3.06)
30-44	1.55 (1.37-1.75)	2.47 (2.24-2.71)
45-64	1.27 (1.13-1.43)	2.03 (1.86-2.23)
65-74	1.43 (1.25-1.62)	1.63 (1.48-1.81)
⩾75	1.0 (ref)	1.0 (ref)

**Race/ethnicity**		

Non-Hispanic white	1.0 (ref)	1.0 (ref)
Non-Hispanic black	0.74 (0.68-0.82)	0.63 (0.59-0.68)
Hispanic	0.74 (0.66-0.83)	0.73 (0.67-0.81)
Other	0.70 (0.62-0.79)	0.75 (0.66-0.84)

**Education**		

Less than high school	1.0 (ref)	1.0 (ref)
High school	1.40 (1.26-1.56)	1.23 (1.13-1.34)
Some college or technical school	1.71 (1.54-1.90)	1.40 (1.28-1.53)
College graduate	1.84 (1.66-2.04)	1.64 (1.50-1.79)

**Body mass index (BMI)**		

Underweight (<18.5)	0.60 (0.42-0.85)	0.83 (0.73-0.94)
Healthy weight (18.5-24.9)	1.0 (ref)	1.0 (ref)
Overweight (25.0-29.9)	1.01 (0.95-1.07)	0.77 (0.73-0.81)
Obese (⩾30.0)	0.68 (0.63-0.73)	0.53 (0.50-0.56)

**Region**		

South	1.0 (ref)	1.0 (ref)
Northeast	1.11 (1.04-1.19)	1.17 (1.10-1.24)
Midwest	1.03 (0.97-1.10)	1.04 (0.99-1.10)
West	1.24 (1.15-1.34)	1.33 (1.25-1.43)

**Employment status/occupational activity**		

Employed, mostly sitting or standing	1.0 (ref)	1.0 (ref)
Employed, mostly walking	1.17 (1.08-1.27)	1.18 (1.10-1.27)
Employed, mostly heavy labor	1.40 (1.29-1.52)	1.69 (1.51-1.91)
Not currently employed	1.15 (1.07-1.24)	1.20 (1.14-1.27)

a Adjusted for all variables shown. Recommended levels of physical activity = participating in 30 minutes per day of moderate-intensity activity on five or more days per week or 20 minutes per day of vigorous-intensity activity on three or more days per week. OR = odds ratio; CI = confidence interval; ref = referent group.
